# Supermicrosurgery in reconstructive surgery: a comprehensive bibliometric analysis

**DOI:** 10.1016/j.jpra.2026.09.001

**Published:** 2026-09-07

**Authors:** Dilip Gosall, Oluwatobi Adegboye, Michalis Hadjiandreou, Gurjinderpal Singh Pahal, Georgios Pafitanis

**Affiliations:** a St John’s College, The University of Cambridge, Cambridge, UK; b St Andrew’s Centre for Plastic Surgery & Burns, Broomfield Hospital, Chelmsford, Essex, UK; c Department of Plastic and Reconstructive Surgery, The Royal London Hospital, Barts Health NHS Trust, London, UK

**Keywords:** Supermicrosurgery, Reconstructive surgery, Bibliometric analysis, Microsurgery, Lymphatic surgery, Plastic surgery

## Abstract

**Background:**

Supermicrosurgery represents an important advancement within the field of reconstructive surgery. This advancement has enabled anastomosis of vessels ≤0.8 mm and has broadened applications in lymphatic surgery, perforator-based reconstruction and technology-based microsurgery. Clinical applications have advanced substantially, but the evolution of the field has not been comprehensively characterised.

**Methods:**

A bibliometric analysis was conducted to assess the global supermicrosurgery literature. A systematic search of PubMed, Web of Science and the Cochrane Library was performed on 1 April 2026 using the search terms 'supermicrosurgery' OR 'supramicrosurgery'. Publications describing clinical, translational, technical, or imaging-related applications within the field of supermicrosurgery were included. Data extracted comprised publication characteristics, citation metrics, anatomical distribution, technique classification, and Oxford Centre for Evidence-Based Medicine levels of evidence. Descriptive analyses of temporal and thematic trends were undertaken.

**Results:**

A total of 175 studies published between 1998 and 2026 were included, accumulating 3578 citations. Publication output increased after 2013 and reached its highest level between 2023 and 2024. The largest domain was lymphatic applications (58.3%), followed by reconstructive applications (30.3%). The most commonly studied anatomical regions were lower limb and combined extremities. There was a marked growth in technology-enabled supermicrosurgery after 2020 and this included robotic and digitally assisted techniques. Level IV observational studies predominated in the literature, accounting for 54.3% of included studies. Japan was the leading contributor followed by South Korea, Italy, and the United States.

**Conclusion:**

Supermicrosurgery has developed from a specialised reconstructive technique into a broad technical framework with expanding applications in lymphatic surgery and technology-assisted surgery. Despite rapid academic growth, the field remains limited by a predominance of lower-quality evidence, highlighting the need for higher-quality comparative and prospective research.

## Introduction

Supermicrosurgery is a rapidly advancing technique within reconstructive surgery, characterised by the anastomosis of vessels typically <0.8 mm in diameter rather than the 1–2 mm defined in conventional microsurgery .^1^^,^^2^ The evolution of supermicrosurgery has expanded the indications and precision of reconstructive operations, such as in lymphoedema surgery or perforator-to-perforator anastomosis .^3^^,^^4^ In particular, supermicrosurgical techniques have transformed the management of complex defects, lymphoedema, and functional reconstruction, reflecting a broader shift towards minimally invasive, physiology-preserving interventions .^5^, ^6^, ^7^

Bibliometric analysis offers a robust methodological approach to quantitatively evaluate the structure and evolution of research within a field, identifying influential publications, key authors, collaborative networks, and thematic trends .^8^ Within reconstructive surgery, such analyses are increasingly valuable for mapping innovation, assessing research impact, and highlighting disparities in evidence generation and dissemination. The growth of supermicrosurgery as an academic discipline has been heterogeneous, with contributions spanning multiple subspecialties, institutions, and geographic regions .^9^

To date, bibliometric analyses have examined specific domains of supermicrosurgery, particularly lymphatic surgery, but no comprehensive bibliometric analysis has evaluated the overall landscape of reconstructive supermicrosurgery .^10^ This study aims to address this gap by systematically analysing the most influential literature in supermicrosurgery, examining patterns in citation metrics, authorship, institutional output, and levels of evidence. By doing so, this analysis will provide insight into the development of the field, identify areas of research strength and evidence gaps, and inform future directions for both clinical practice and academic investigation.

## Materials and methods

### Study design

This study was designed as a systematic bibliometric analysis of the supermicrosurgery literature. The study was conducted and reported in accordance with the PRISMA 2020 statement, where applicable. This analysis aimed to characterise the size, evolution, structure, and methodological profile of supermicrosurgery rather than to perform pooled outcome synthesis. Hence, the study combined bibliometric, descriptive, and thematic classification methods.

### Search strategy

A structured literature search was conducted in PubMed, Web of Science, and the Cochrane Library on the 1st of April 2026. The following search strategy was applied to the title and abstract fields: (“supermicrosurgery” OR “supramicrosurgery”). No restrictions were placed on publication date, language, or study design. The search strategy was intentionally focused on the key terms - “supermicrosurgery” OR “supramicrosurgery” - rather than based on controlled vocabulary terms such as MeSH to keep the search broad yet relevant. This search strategy was required to support the identification and analysis of literature that utilised the key terms and the evolution of key terms across time.

### Eligibility criteria

Eligibility criteria were defined a priori. Supermicrosurgery was defined as procedures involving vessels ≤0.8 mm in diameter where explicitly specified, or as described by the study authors. The inclusion criteria were description of supermicrosurgery or microsurgical techniques; studies involving humans or clinical, translational, or technical applications; and studies that demonstrated clinical, operative, or imaging relevance to supermicrosurgery.

### Study selection and data extraction

Following preliminary title and abstract filtering within each database, records were exported and merged in Google Sheets, where duplicates were identified and removed manually. Titles and abstracts were assessed independently by two reviewers (D.G. and O.A.), followed by full-text review against predefined eligibility criteria. Discrepancies were resolved by consensus.

Data extraction was performed using a standardised proforma in Google Sheets by two reviewers, with discrepancies resolved by consensus. Extracted data included bibliometrics, study characteristics, and clinical information such as supermicrosurgical technique and outcomes. Comparator characteristics were categorised as no comparator (single-arm), within-subject comparison, between-group comparison, implicit/indirect comparison, or not applicable (such as a review or technical studies). Level of Evidence was ascertained using Oxford Centre for Evidence-Based Medicine (OCEBM) (.^11^).

### Study classification framework

Studies were classified according to their focus across clinical application and conceptual domains to enable consistent analysis. Classification was based a priori on the dominant emphasis of each study, acknowledging that individual studies may span multiple areas, and was divided into primary and secondary classification.

Primary classification was subdivided into three categories and used to describe the clinical and anatomical distribution of supermicrosurgical applications:

● Clinical conditions such as lymphatic, reconstructive, oncological, trauma, and other.
● Anatomical region such as upper limb, lower limb, head and neck or multi-site.
● Technique groups such as lymphatic, reconstructive, technology-enabled, and nerve.

Secondary classification was subdivided into four categories and used to analyse temporal trends and the evolving use of supermicrosurgery across domains.

● Conceptual / Technical.
● Reconstructive.
● Lymphatic.
● Technology-enabled.

### Data analysis and visualisation

Descriptive statistics were produced using Google Sheets. Continuous bibliometric variables were summarised using medians and interquartile ranges where appropriate. Categorical variables were summarised as counts and percentages. Bibliometric analysis was performed across all included studies and was not restricted to highly cited publications, allowing evaluation of the overall structure of the field.

Variables included total number of studies, citation count, median citations per study, citations per year, open access status, publication year, geographic origin, and journal distribution. Citation velocity was calculated as citations per year (CPY), defined as total citations divided by years since publication (with reference to 2026).

Graphical outputs included temporal trend analyses and domain-based distributions. Anatomical representation was illustrated using a schematic “supermicrosurgical homunculus,” with colour intensity reflecting relative research activity.

### Ethical considerations

As this study was a bibliometric analysis of previously published literature, formal ethical approval and patient consent were not required. This approach is consistent with standard reporting in published bibliometric surgical studies .^12^

## Results

### Overview of included studies

A total of 484 records were identified, of which 241 remained after preliminary title and abstract filtering. Following removal of 24 duplicates, 217 unique records underwent screening. Forty-two full-text studies were excluded, resulting in 175 studies included in the final analysis (Fig. 1 and Table 1).

**Fig. 1:**
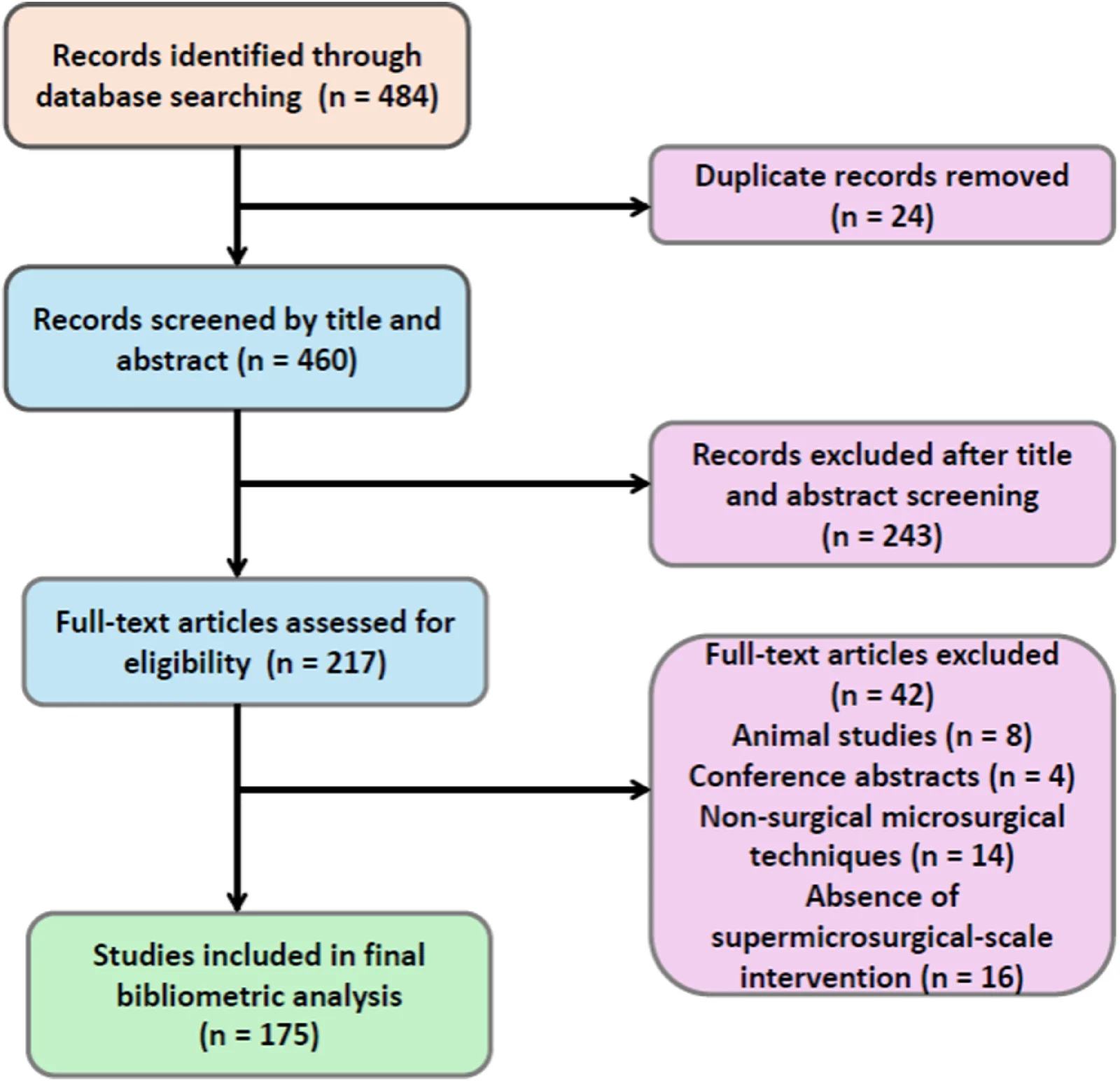
PRISMA flow diagram illustrating study selection and inclusion in the bibliometric analysis (n = 175).

**Table 1:** Characteristics of studies included in the final bibliometric analysis (n = 175).

Bibliometric Characteristics	
Total number of studies	175
Total citation count	3578
Median citations per study	8 (IQR 3–24)
Median citations per year (CPY)	1.5 (IQR 0.5–3.5)
Open access studies	26 (14.9%)
Publication years	1998–2026; IQR 2015–2023

Study Characteristics, n (%)	
Level of evidence (OCEBM)	Level I 11 (6.3%) Level II 2 (1.1%) Level III 32 (18.3%) Level IV 95 (54.3%) Level V 35 (20%)
Comparator Characteristics	No comparator (single-arm) 112 (64.0%) Within-subject comparison 30 (17.1%) Between-group comparison 20 (11.4%) Implicit/indirect comparison 8 (4.6%) N/A (reviews/technical) 5 (2.9%)

Clinical Characteristics, n (%)	
Anatomical distribution Percentages may not total 100% as less frequently represented or unspecified anatomical categories are not displayed.	Lower limb: 46 (26.3%) Combined extremities: 41 (23.4%) Multi-site: 31 (17.7%) Upper limb: 28 (16.0%) Head and neck: 15 (8.6%) Trunk/breast 8 (4.6%)
Clinical indications	Lymphatic disease: 102 (58.3%) Reconstructive defects: 53 (30.3%) Oncological reconstruction: 8 (4.6%) Trauma: 8 (4.6%) Other: 4 (2.3%)

Dominant Supermicrosurgical Domains n (%)	
Technique groups Studies were assigned to a single dominant supermicrosurgical domain based on the primary surgical or technological focus of each publication.	Lymphatic: 85 (48.6%) Reconstructive (flap-based): 56 (32.0%) Technology-enabled: 31 (17.7%) Nerve: 2 (1.1%) Broad/Conceptual: 1 (0.6%)

**Fig. 2:**
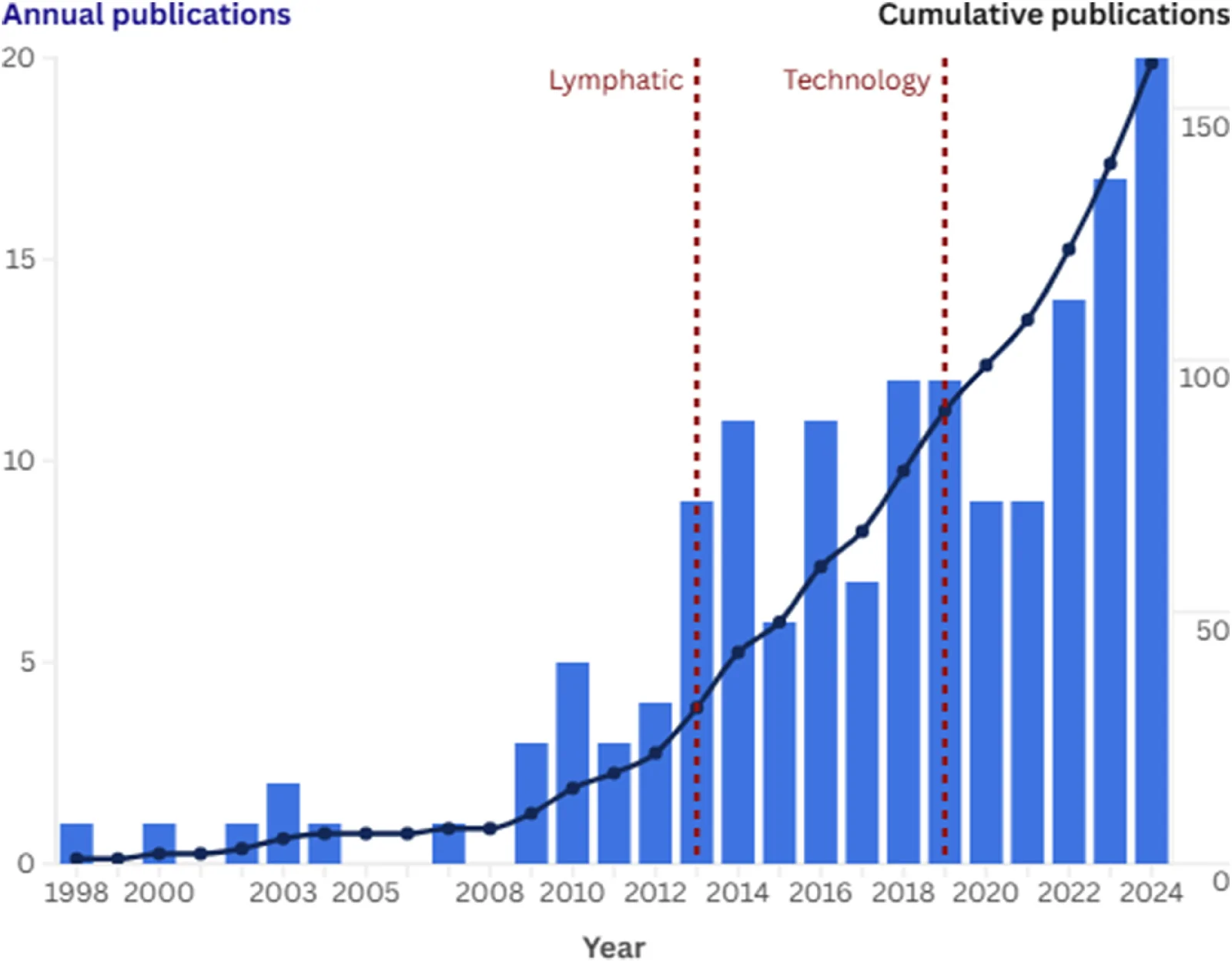
Annual and cumulative publication output in supermicrosurgery (1998 – 2026).

### Temporal growth of the field

The included 175 studies were published between 1998 and 2026. Annual publication output was minimal before 2010, followed by a gradual increase between 2010 and 2013. Publication volume then increased steadily, with a marked acceleration after 2020. The highest annual output was observed in 2024, approaching 20 publications, with similarly high output in 2023, reflecting the rapid expansion of supermicrosurgery as an established and evolving field (Fig. 2).

### Applications

Analysis of the distribution of secondary classification of supermicrosurgery publications demonstrated a shift in research focus. Initially, supermicrosurgery was primarily used as an advanced reconstructive technique. From 2010 to 2016, there was a rapid increase in published studies that described the use of supermicrosurgery in lymphatic surgery, particularly lymphaticovenous anastomosis (LVA). Between 2017 and 2021, there were a number of studies published relevant to reconstructive and lymphatic applications of supermicrosurgery, however more contemporary studies (2022 onwards) have reported on technology-enabled applications including robotically and digitally assisted techniques. Quantitatively, lymphatic surgery represented the largest category, followed by reconstructive applications, whereas studies on integrating new technology only demonstrated a marked increase after 2020 (Fig. 3, Fig. 4). Overall, these findings demonstrate a transition from reconstructive innovation to lymphatic clinical expansion, followed by increasing technological integration.

**Fig. 3:**
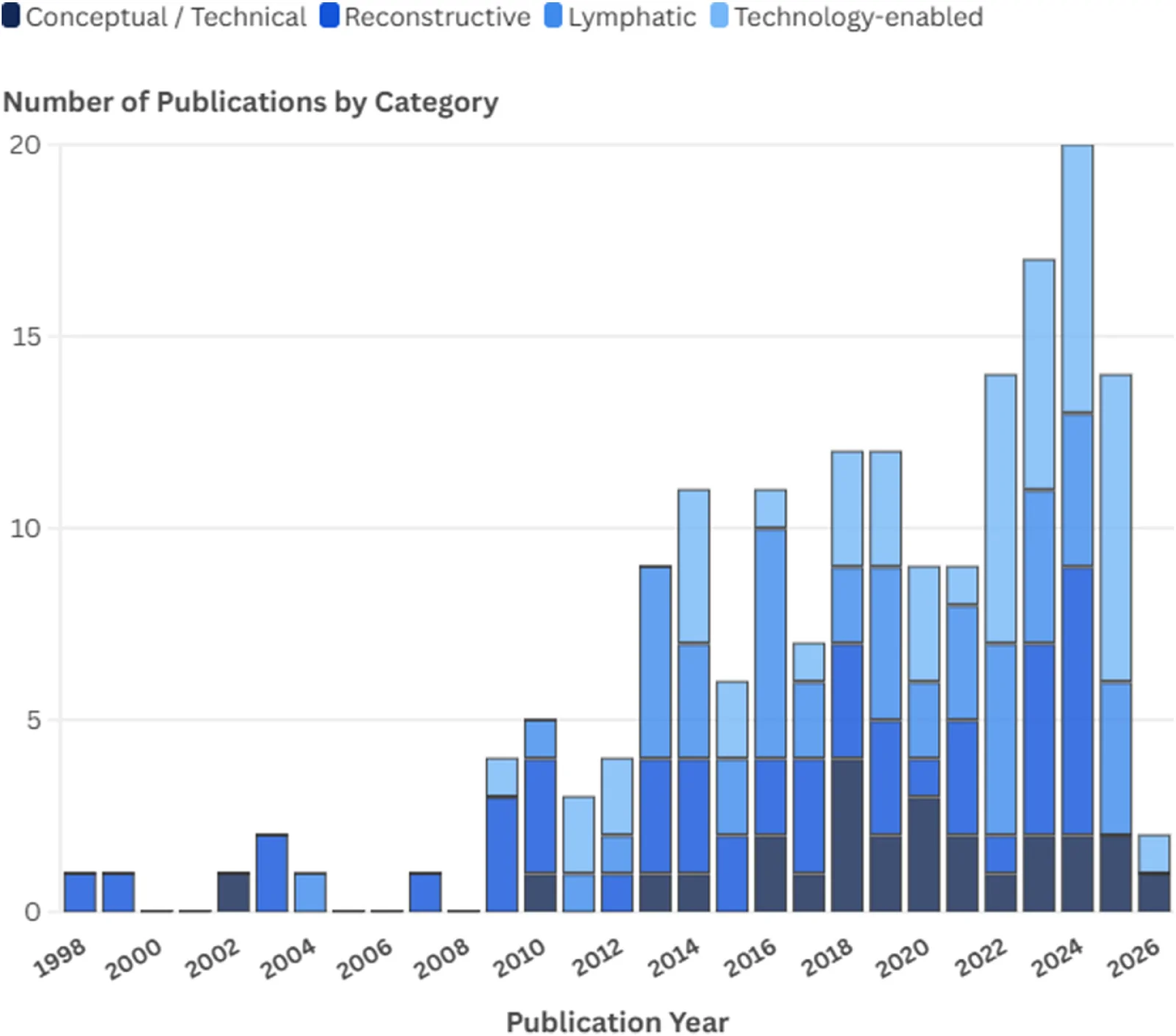
Annual publication output by supermicrosurgical category (1998–2026).

**Fig. 4:**
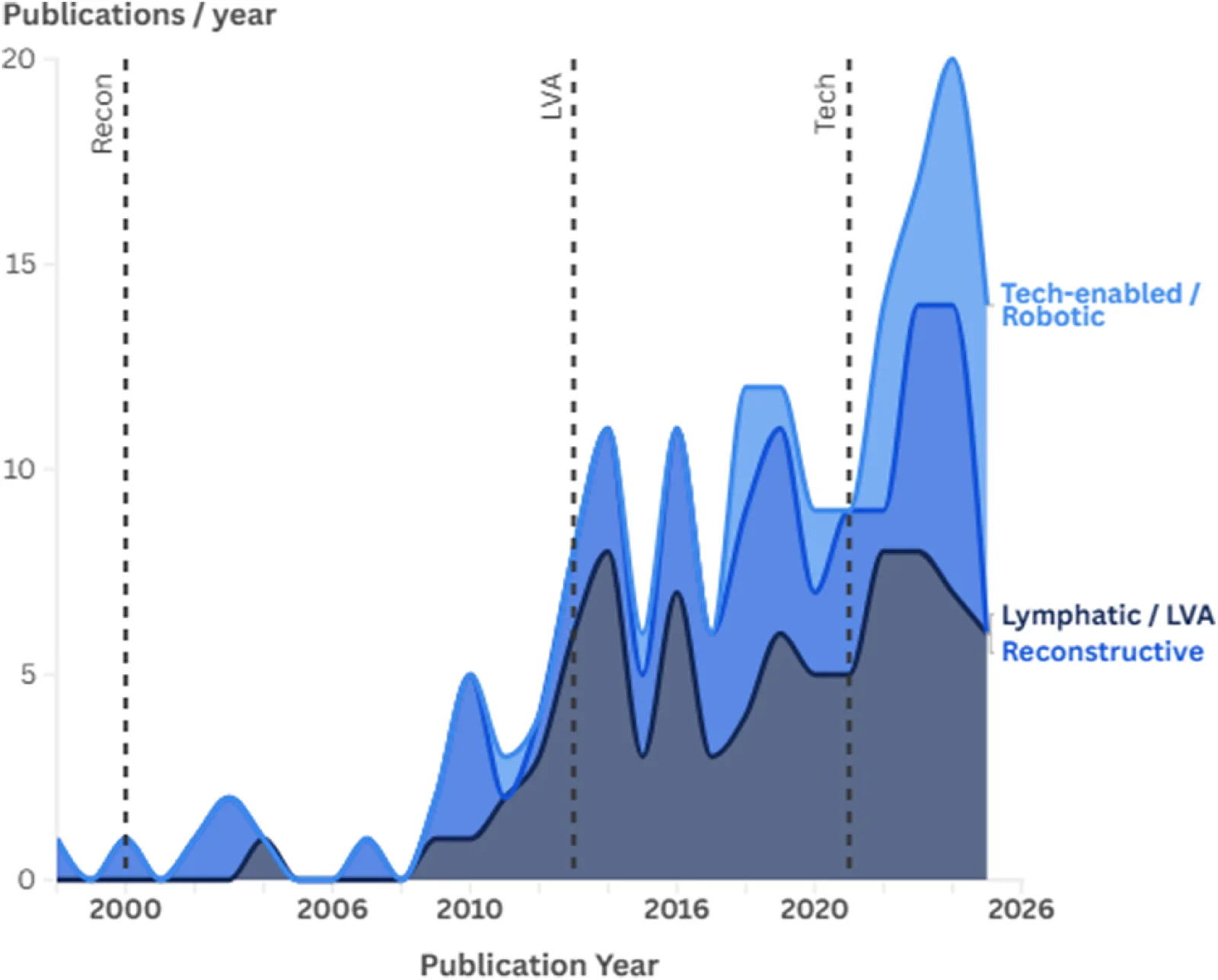
Temporal evolution of supermicrosurgery across reconstructive, lymphatic and technology-enabled domains (1998–2026).

**Fig. 5:**
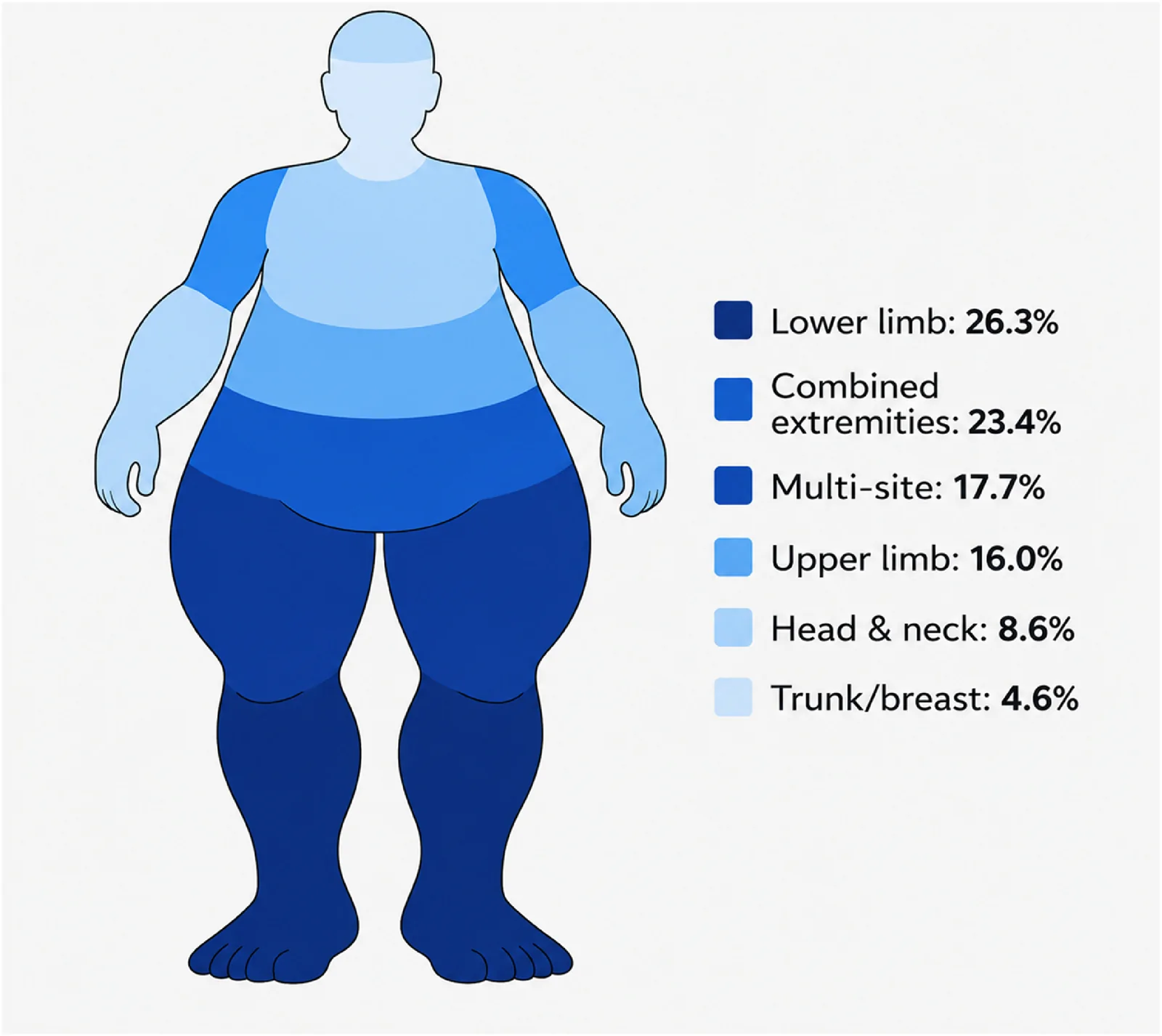
Anatomical distribution of supermicrosurgical applications illustrated using a supermicrosurgical homunculus.

**Fig. 6:**
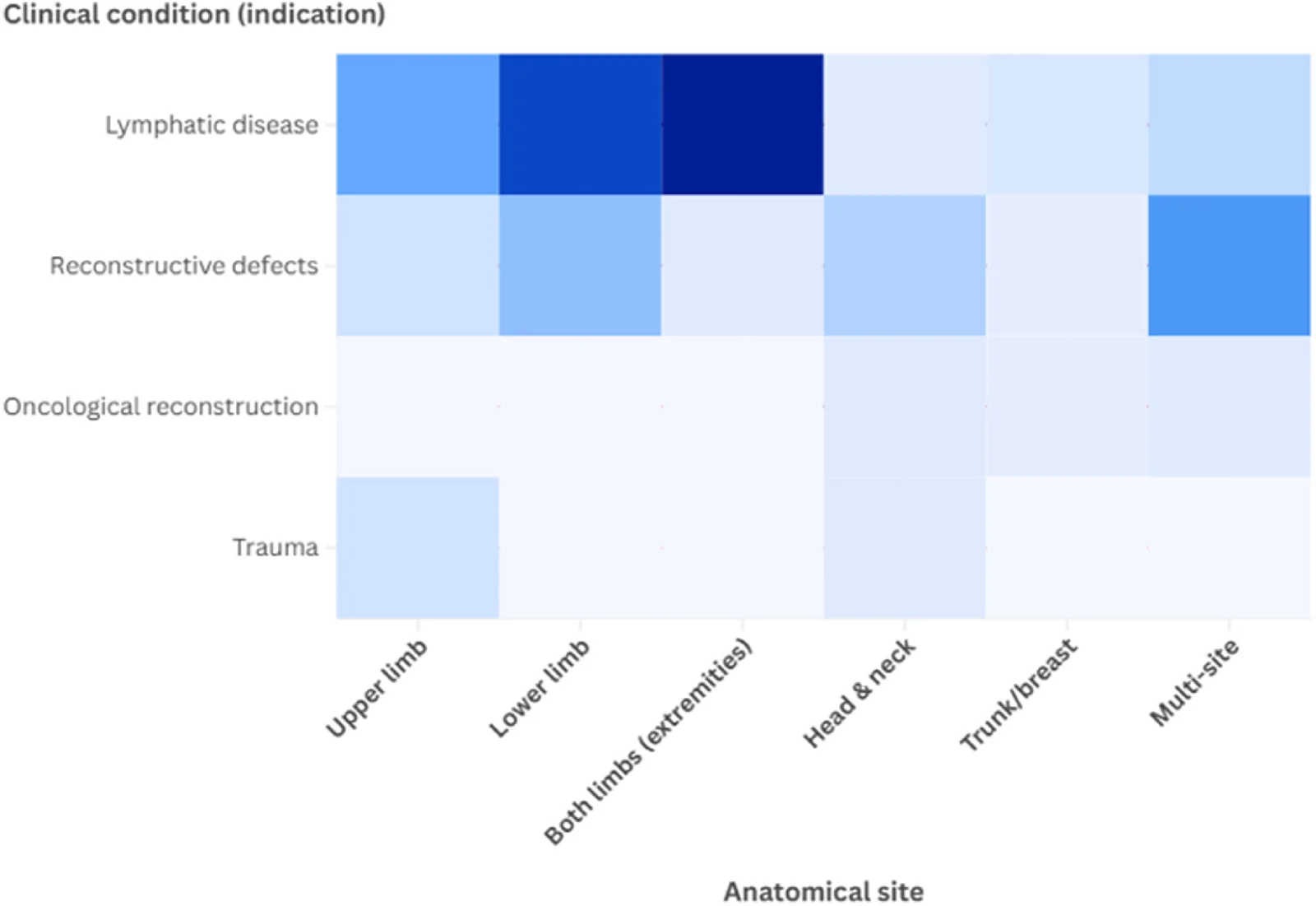
Heat map illustrating the relationship between clinical condition and anatomical site in supermicrosurgery publications. Colour intensity represents the number of publications, with darker shading indicating higher frequency.

### Anatomical and clinical distribution

Supermicrosurgical applications were most frequently reported in the extremities. Head and neck and trunk applications were less commonly represented. Fig. 5 illustrates the anatomical distribution of supermicrosurgical research activity using a supermicrosurgical homunculus. Supermicrosurgery for lymphatic disease was predominantly reported in the extremities, particularly in studies involving both limbs, followed by the lower limb. In contrast, reconstructive applications are more widely distributed across anatomical regions, with notable representation in multi-site studies and head and neck reconstruction. Oncological reconstruction exhibits a site-specific pattern, primarily involving the head and neck and trunk/breast regions. Trauma-related applications are largely confined to the upper limb, with limited representation in other anatomical sites. The relationship between clinical condition and anatomical site is shown in Fig. 6.

### Citation profile and study characteristics

The total citation count of the included studies was 3578, with a median of 8 citations per article (CPA) and 1.5 citations per year (CPY). The highly cited studies predominantly discussed early reconstructive innovations or foundational lymphatic studies. In contrast, higher CPY was observed in more recent technology-related studies, indicating growing contemporary influence. These findings demonstrate a divergence between historical impact and current academic influence within the field. Table 2 displays the characteristics for the 10 most cited studies included in this review, while Supplementary Table 1 summarises all included studies. Substantial variation in CPY is observed across jourmals, reflecting differences in study design, innovation, and target audience (Fig. 7).

**Table 2:** Top 10 most-cited publications in supermicrosurgery.

Rank	Year	First Author	Citations	CPY	Study Type	Primary Domain	Key Contribution
1	2010	Koshima I et al.	188	11.75	Review	Broad	Foundational perforator + supermicrosurgery framework
2	2020	van Mulken et al.	156	26.00	RCT (pilot)	Tech-enabled	First-in-human robotic LVA
3	2004	Koshima I et al.	149	6.77	Clinical study	Lymphatic	Early LVA for advanced lymphedema
4	2014	Yamamoto T et al.	143	11.92	Case series	Lymphatic	MILS technique (ICG-guided multisite LVA)
5	1998	Koshima I et al.	139	4.96	Clinical study	Reconstructive	Paraumbilical perforator flap innovation
6	2021	Hong JP et al.	125	25.00	Review	Broad	Modern lower limb supermicrosurgery synthesis
7	2009	Hong JP	125	7.35	Clinical series	Reconstructive	Lower limb perforator-to-perforator evolution
8	2013	Hong JP et al.	123	9.46	Clinical series	Reconstructive	SCIP flap + supermicrosurgery refinement
9	2016	Mihara M et al.	112	11.20	Cohort study	Lymphatic	Multisite LVA with NECST classification
10	2010	Hong JP & Koshima I	109	6.81	Clinical series	Reconstructive	Perforator as recipient vessel concept

The evidence base was predominantly observational. Single-arm studies were the most common comparator category (64.0%). Broadly, there was limited use of comparative designs between groups or treatments (11.4%). The most common Level of Evidence of included studies was Level IV (54.3%) followed by Level V (20%). Overall, the literature reflects limited studies with high level comparative evidence.

The studies were published in a wide range of journals. Most journals contributed low publication volume. However, core plastic surgery journals, such as the Journal of Plastic, Reconstructive & Aesthetic Surgery (JPRAS) and Plastic and Reconstructive Surgery (PRS), had the overall highest citation count. Some of the higher impact journals, such as Nature Communications or Radiology, demonstrated a higher CPY but lower publication volume. Substantial variation in CPY is observed across journals, reflecting differences in study design, innovation, and target audience.

Japan is the dominant contributor to the supermicrosurgery literature (33%), followed by South Korea (10%), Italy (10%), the United States of America (9%), and China (7%). Research output by country demonstrated clear geographic clustering, with most studies published from East Asia and select high income Western countries (Fig. 8). Contribution from low- and middle-income regions remains limited, likely reflecting the resource intensive nature of supermicrosurgery techniques. Supermicrosurgery is geographically concentrated, likely reflecting the presence of high-volume specialised centres which are clinical and academic leaders in the field.

**Fig. 7:**
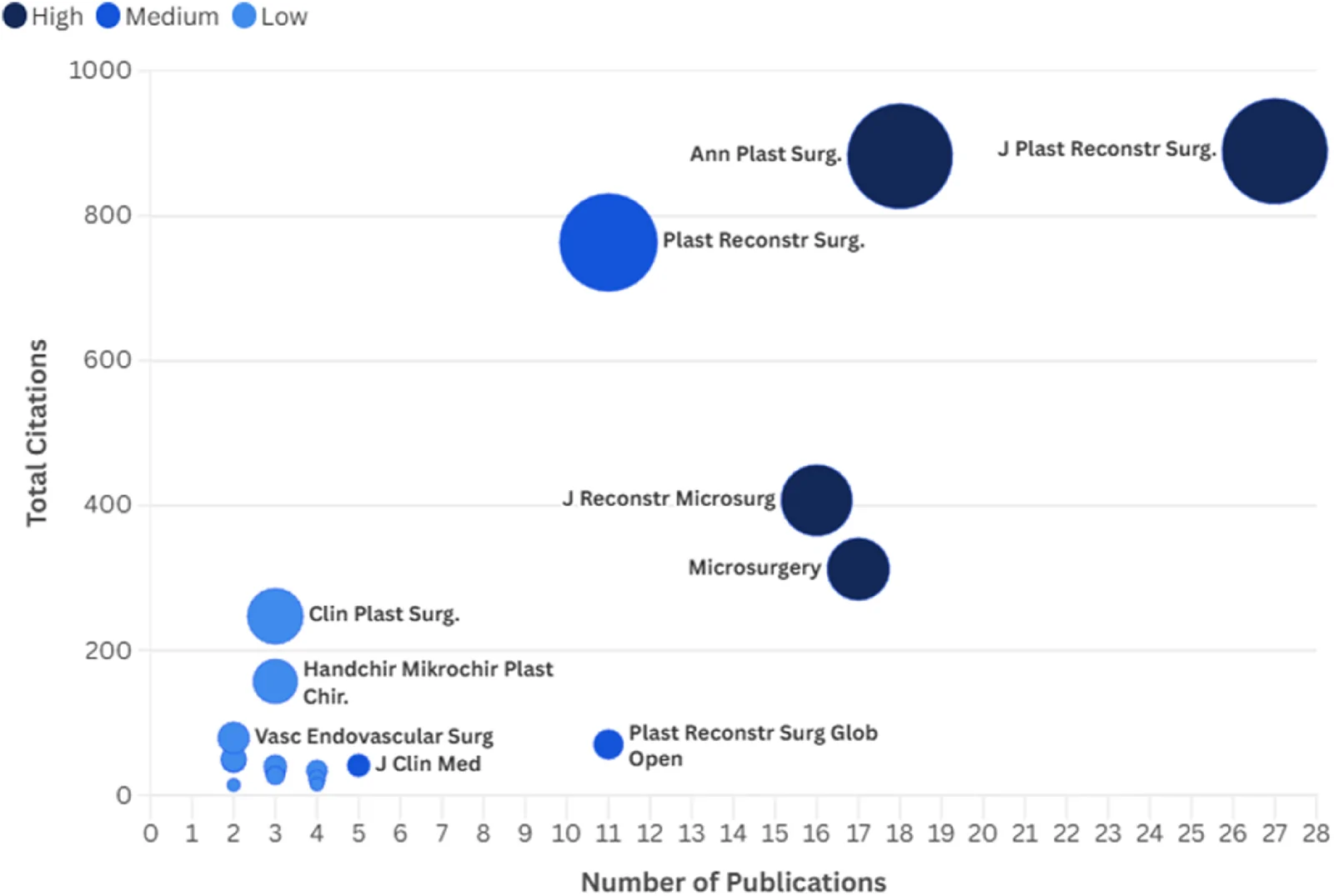
Journal productivity and citation impact of supermicrosurgery publications. The x-axis indicates the number of publications, the y-axis represents total citations, bubble size reflects mean citations per publication, and bubble colour indicates publication-volume category.

**Fig. 8:**
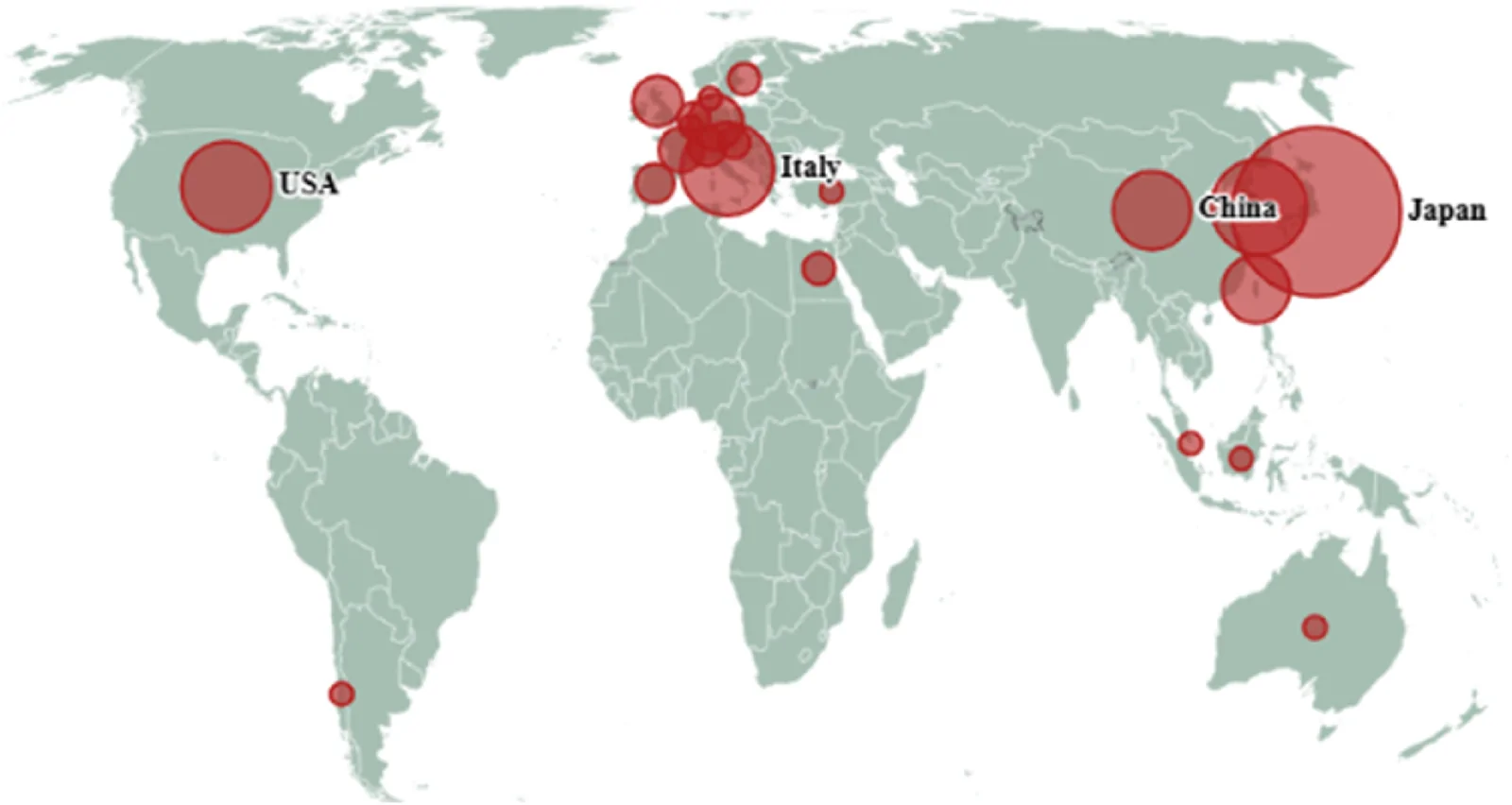
Global distribution of supermicrosurgery publications. Bubble size represents publication volume by country.

## Discussion

### Principal findings

Previous bibliometric studies have characterised publication growth and geographic distribution, while more recent work has focused on lymphatic surgery; however, none has comprehensively examined how supermicrosurgery has evolved across multiple clinical domains and conceptual frameworks .^10^^,^^13^ This bibliometric analysis presents data that delineates the progressive transition of supermicrosurgery from primarily reconstructive applications toward lymphatic surgery, with more recent expansion into technology enhanced approaches. The predominance of lymphatic applications and the emergence of technology-assisted techniques display that supermicrosurgery applications are evolving towards broader use.

### Evolution of supermicrosurgery across clinical domains

The temporal patterns observed in this study appear consistent with trends described in recent literature. A 2026 bibliometric analysis of lymphatic surgery describes a shift from reductive procedures toward physiologic reconstruction using supermicrosurgery techniques such as LVA .^10^ This is reflected in the present analysis, where lymphatic applications represent the most prominent and rapidly expanding domain since approximately 2010.

Both analyses highlight that lymphatic procedures now form a central component of contemporary supermicrosurgery practice, while the literature remains dominated by lower level and quality evidence. These findings suggest that the expansion of supermicrosurgery has been closely linked to its adoption within lymphatic surgery, where it has enabled new physiologically based treatment approaches.

### Relationship to reconstructive supermicrosurgery

Reconstructive applications represent the earliest and most technically foundational use of supermicrosurgery. The systematic review by Lo Torto et al. .^3^ reports that perforator-to-perforator supermicrosurgery flaps demonstrate acceptable complication rates comparable to conventional microsurgical techniques, while also highlighting limitations in overall study quality .^3^ This aligns with the present findings, in which reconstructive applications dominate early phases of the literature, remain consistently represented over time, and are supported primarily by observational studies. These observations suggest that reconstructive supermicrosurgery continues to underpin the field, even as newer applications have expanded.

### Emergence of technology-enabled applications

A notable recent development is the increasing representation of technology-enabled supermicrosurgery, particularly after 2020. This trend is supported by contemporary literature. A systematic review by Brown et al.^14^ reports that robotic-assisted supermicrosurgery may improve precision and reduce microtrauma, with high reported anastomotic patency rates, although operative times are initially longer and decrease with experience .^14^ In this context, the observed increase in technology-enabled studies may reflect early adoption of enabling technologies, particularly within lymphatic procedures such as LVA.

### Conceptual implications

Taken together, the findings suggest that supermicrosurgery may be more consistently defined by its technical characteristics than by any single disease or anatomical domain. The distribution of studies across reconstructive, lymphatic, and emerging technological domains supports the view that supermicrosurgery functions as a technique-based approach with applications across multiple subspecialties. Rather than representing a single clinical discipline, it may be more appropriate to consider supermicrosurgery as a technical framework that enables diverse surgical applications.

### Geographic variation

Geographic variation in publication output suggests that supermicrosurgery has developed unevenly across regions. High-volume contributions from countries such as Japan, Italy, and South Korea likely reflect established microsurgical expertise, investment in specialised resources and well-developed training pathways. For example, Kono Seisakusho, a Japanese company, has made significant contributions to the development of microsurgical instrumentation, while Symani, an Italian robotic microsurgery platform, represents one of the pioneering technologies in robotic supermicrosurgery .^14^^,^ ^15^ To facilitate the wider adoption of supermicrosurgery and improve global equity, highly specialised centres with established expertise should be encouraged to act as training hubs. This may include publishing and sharing models of service development, outreach programmes to support non-specialist centres, and the development of observerships and fellowships that provide surgeons worldwide with opportunities for specialist training. Overall, the geographic variation suggests that institutional expertise, technological innovation, and investment in training infrastructure have played a key role in the development of contemporary supermicrosurgery.

### Methodological considerations and future directions

This study provides original bibliometric and clinical data describing the evolution of supermicrosurgery; however, several limitations should be acknowledged. Several potential sources of selection bias should be considered. For example, a keyword-based search strategy may exclude relevant studies not explicitly labelled as supermicrosurgery. Furthermore, bibliometric analysis reflects publication trends rather than clinical outcomes. Citation metrics reflect academic influence rather than methodological quality or clinical effectiveness. This analysis included clinical data relevant to supermicrosurgery such as anatomical site and clinical condition, however, the heterogeneity in study design limits direct comparison across categories. Despite ongoing expansion, the evidence base remains limited in methodological quality. Consistent with prior bibliometric analyses, including the 2026 study by Al Qurashi et al., the literature is dominated by case series and lower levels of evidence, with relatively few comparative or randomised studies .^10^ Similarly, reconstructive literature has been noted to lack high-quality comparative data, limiting the strength of conclusions regarding clinical effectiveness .^3^ This highlights the need for prospective comparative studies, standardised outcome reporting, and further evaluation of emerging technologies. This study provides a broad bibliometric overview of supermicrosurgery, with a focus on terminology usage and technique-based classification across multiple domains.

## Conclusion

Supermicrosurgery has evolved from an extension of microsurgery in complex reconstructive surgery into its own field with expanding applications across several aetiologies including lymphatic disease and trauma. The integration of robotics, imaging, and digital planning tools suggests that the field is likely to continue evolving, with potential for broader adoption as technical barriers are reduced. Existing literature has examined supermicrosurgery either through narrative historical reviews or through disease-specific bibliometric analyses, particularly within lymphatic surgery. However, there is a paucity in high- quality prospective studies, such as randomised controlled trials or cohort studies, which reliably report on the benefits and considerations of supermicrosurgery, especially compared to traditional microsurgery.

## Funding statement

The authors received no specific funding for this work.

## Ethics approval statement

Ethical approval was not required for this bibliometric analysis of previously published literature.

## Patient consent statement

Patient consent was not required.

## Permission to reproduce material from other sources

No copyrighted material from other sources requiring permission has been reproduced.

## Declaration of generative AI and AI-assisted technologies in the manuscript preparation process

During the preparation of this work, the authors used OpenAI ChatGPT to assist in refining the presentation of **Figure 5**, which was based on an original author-created drawing. The authors reviewed and approved the final figure and take full responsibility for the content of the published work.

## Declaration of competing interest

The authors declare no conflicts of interest.

## Data Availability

The data supporting the findings of this study are available from the corresponding author upon reasonable request.

## Appendix Supplementary materials

Supplementary Table 1: Citation profile and characteristics of included studies.
