# Supplementary material for: Supermicrosurgery in reconstructive surgery: a comprehensive bibliometric analysis
Source: JPRAS Open. 2026 Sep 7;52:673–84. doi: 10.1016/j.jpra.2026.09.001 (PMC13731123; doi:10.1016/j.jpra.2026.09.001)
Supplement: Supplementary file 1 [file mmc1.docx]

| **Year** | **First Author** | **Citations** | **CPY** | **Study Type** | **Primary Domain** | **Key Contribution** |
| --- | --- | --- | --- | --- | --- | --- |
| 2010 | Koshima I et al. | 188 | 11.75 | Review | Broad | Foundational perforator + supermicrosurgery framework |
| 2020 | van Mulken et al. | 156 | 26.00 | RCT (pilot) | Tech-enabled | First-in-human robotic LVA |
| 2004 | Koshima I et al. | 149 | 6.77 | Clinical study | Lymphatic | Early LVA for advanced lymphedema |
| 2014 | Yamamoto T et al. | 143 | 11.92 | Case series | Lymphatic | MILS technique (ICG-guided multisite LVA) |
| 1998 | Koshima I et al. | 139 | 4.96 | Clinical study | Reconstructive | Paraumbilical perforator flap innovation |
| 2021 | Hong JP et al. | 125 | 25.00 | Review | Broad | Modern lower limb supermicrosurgery synthesis |
| 2009 | Hong JP | 125 | 7.35 | Clinical series | Reconstructive | Lower limb perforator-to-perforator evolution |
| 2013 | Hong JP et al. | 123 | 9.46 | Clinical series | Reconstructive | SCIP flap + supermicrosurgery refinement |
| 2016 | Mihara M et al. | 112 | 11.20 | Cohort study | Lymphatic | Multisite LVA with NECST classification |
| 2010 | Hong JP & Koshima I | 109 | 6.81 | Clinical series | Reconstructive | Perforator as recipient vessel concept |
| 2011 | Yamamoto T et al. | 94 | 6.27 | Case report (2 cases) | Lymphatic | Multi-site LVA for severe lymphorrhea in primary lower limb and genital lymphedema |
| 2014 | Ito R et al. | 85 | 7.08 | Review article | Broad/conceptual | Overview of LVA and vascularized lymph node transfer for lymphedema treatment |
| 2013 | Yamamoto T et al. | 84 | 6.46 | Case series | Lymphatic | Modified side-to-end LVA with anti-reflux technique for lower limb lymphedema |
| 2016 | Suh HS et al. | 83 | 8.3 | Retrospective cohort study | Reconstructive | Angiosome-guided perforator-to-perforator reconstruction for diabetic foot wounds |
| 2008 | Narushima M et al. | 66 | 3.67 | Animal study | Technology-enabled | Intravascular stenting technique for safer supermicrosurgical anastomosis |
| 2018 | Hong JPJ et al. | 77 | 9.63 | Review article | Broad/conceptual | Overview of supermicrosurgical principles and clinical applications |
| 2014 | Yamamoto T et al. | 77 | 6.42 | Comparative case series | Technology-enabled | ICG-guided near-infrared microscope-integrated LVA |
| 2016 | Yamamoto T et al. | 76 | 7.6 | Case report | Lymphatic | Complete lymph flow reconstruction using VLNT with lymphaticolymphatic anastomosis |
| 2016 | Masia J et al. | 74 | 7.4 | Retrospective case series | Lymphatic | Combined LVA and autologous lymph node transplantation for breast cancer-related lymphedema |
| 2016 | Chen WF et al. | 71 | 7.1 | Prospective clinical study | Technology-enabled | ICG lymphography assessment of lymphatic reconstruction efficacy |
| 2003 | Koshima I et al. | 70 | 3.04 | Case report | Reconstructive | Medial plantar perforator flap using supermicrosurgical anastomosis |
| 2015 | Chen WF et al. | 68 | 6.18 | Prospective case series | Technology-enabled | “Octopus” multi-lymphatic-to-single-venule LVA technique |
| 2024 | Xie Q et al. | 52 | 26 | No abstract/editorial | Broad/conceptual | Conceptual perspective on future neuro-supermicrosurgery applications |
| 2018 | Badash I et al. | 65 | 8.13 | Review article | Broad/conceptual | Historical overview, applications, training, and future of supermicrosurgery |
| 2014 | Yamamoto T et al. | 63 | 5.25 | Case series | Technology-enabled | Navigation-guided ICG-assisted lymphatic supermicrosurgery |
| 2023 | Innocenti M et al. | 61 | 20.33 | First-in-human feasibility case report | Technology-enabled | First robot-assisted perforator-to-perforator free flap reconstruction |
| 2022 | van Mulken TJM et al. | 58 | 14.5 | Pilot randomized controlled trial | Technology-enabled | One-year outcomes of robot-assisted LVA for breast cancer-related lymphedema |
| 2014 | Choi DH et al. | 57 | 4.75 | Case series | Reconstructive | Thin SCIP flap for facial reconstruction using supermicrosurgery |
| 2019 | Yamamoto T | 56 | 8 | Review article | Broad/conceptual | Overview of onco-reconstructive supermicrosurgery integrating LVA, perforator-to-perforator flaps, and composite reconstruction |
| 2018 | Yang JC et al. | 56 | 7 | Retrospective cohort study | Technology-enabled | ICG-based intraoperative identification of functional lymphatic vessels for LVA |
| 2014 | Yamamoto T et al. | 53 | 4.42 | Case series | Lymphatic | Sequential multi-lymphatic-to-single-venule LVA for lower limb lymphedema |
| 2012 | Mihara M et al. | 52 | 3.71 | Case series | Technology-enabled | ICG- and AccuVein-assisted minimally invasive “scarless” LVA for early lower limb lymphedema |
| 2010 | Demirtas Y et al. | 50 | 3.13 | Retrospective comparative case series | Lymphatic | Comparison of LVA and lymphaticovenous implantation for primary and secondary lower limb lymphedema |
| 2018 | Visconti G et al. | 48 | 6 | Multicenter retrospective cohort study | Lymphatic | Recipient venule flow-dynamic classification and correlation with LVA outcomes |
| 2022 | Jang S et al. | 46 | 11.5 | Retrospective study | Technology-enabled | CEUS microbubble lymphatic mapping before LVA for upper limb lymphedema |
| 2011 | Mihara M et al. | 45 | 3 | Case report | Technology-enabled | Fluorescence-guided LVA for post-oncologic facial/head-and-neck lymphedema |
| 2017 | Gentileschi S et al. | 44 | 4.89 | Case series | Technology-enabled | Healthy-limb mirroring with ICG lymphography for preoperative upper limb lymphatic mapping |
| 2017 | Poumellec MA et al. | 41 | 4.56 | Retrospective cohort study | Lymphatic | Stepped LVA strategy for secondary upper limb lymphedema after breast cancer treatment |
| 2016 | Park JE et al. | 41 | 4.1 | Review article | Broad/conceptual | Overview of advances and innovations in microsurgery including LVA, perforator flaps, chimeric flaps, and freestyle techniques |
| 2014 | Yamamoto T et al. | 40 | 3.33 | Case report | Lymphatic | Ladder-shaped multi-side-to-side LVA for lower limb lymphedema |
| 2013 | Yamamoto T et al. | 39 | 3 | Technical report / case report | Lymphatic | Split intravascular stent technique to facilitate side-to-end LVA |
| 2013 | Todokoro T et al. | 39 | 3 | Case series | Lymphatic | LVA for treatment of post-surgical pelvic lymphocele |
| 2016 | Suh HS et al. | 38 | 3.8 | Review article | Broad/conceptual | Review of supermicrosurgical strategies for diabetic foot reconstruction |
| 2024 | von Reibnitz D et al. | 38 | 19 | Case series | Technology-enabled | Two-year single-centre experience with robotic-assisted lymphatic reconstruction |
| 2019 | Gentileschi S et al. | 37 | 5.29 | Prospective case series | Technology-enabled | SPECT/CT and fusion ultrasound targeting for lymphatic surgery |
| 2012 | Mihara M et al. | 35 | 2.5 | Case report | Technology-enabled | ICG lymphography-guided LVA for cancer-related lymphedema |
| 2019 | Giacalone G et al. | 35 | 5 | Case series | Technology-enabled | ICG-guided LVA for recurrent lymphocele and severe lymphorrhea |
| 2016 | Gennaro P et al. | 34 | 3.4 | Retrospective cohort study | Lymphatic | 36-month outcomes of LVA for lymphedema |
| 2010 | Kim CY et al. | 33 | 2.06 | Case series | Reconstructive | Peroneal/soleus perforator-based free flap reconstruction for ischemic foot defects |
| 2012 | Kim CY et al. | 32 | 2.29 | Comparative study | Reconstructive | Supercharged latissimus dorsi perforator flap reconstruction for ischemic lower limb defects |
| 2013 | Schirmer S et al. | 31 | 2.38 | Case series | Reconstructive | Supramicrosurgical flap reconstruction for diabetic foot ulcers to prevent amputation |
| 2022 | Visconti G et al. | 30 | 7.5 | Case series | Technology-enabled | Multimodality imaging-guided LVA for advanced peripheral lymphedema |
| 2016 | Yamamoto T et al. | 29 | 2.9 | Case series | Lymphatic | Prophylactic efferent lymphatic vessel-to-vein anastomosis for subclinical lymphedema |
| 2016 | Iida T et al. | 28 | 2.8 | Pilot study | Reconstructive | Supermicrosurgical end-to-side anastomosis for head and neck free flap reconstruction |
| 2018 | Mihara M et al. | 28 | 3.5 | Prospective observational study | Technology-enabled | Ultrasound classification of lymphatic sclerosis to guide optimal LVA site selection |
| 2021 | Gupta N et al. | 28 | 5.6 | Systematic review | Lymphatic | Systematic review of LVA outcomes for upper extremity lymphedema |
| 2020 | Tsai PL et al. | 26 | 4.33 | Retrospective cohort study | Lymphatic | Analysis of lymphovascular characteristics and anastomotic configuration in LVA |
| 2018 | Chen WF | 25 | 3.13 | Technical review | Lymphatic | Practical guidance for starting lymphaticovenular anastomosis for lymphedema |
| 2013 | Ayestaray B et al. | 25 | 1.92 | Case series | Lymphatic | π-shaped LVA for head and neck lymphedema |
| 2011 | Matsumura N et al. | 25 | 1.67 | Technical training model | Technology-enabled | Practice card model for basic supermicrosurgery training |
| 2021 | Yang JC et al. | 24 | 4.8 | Retrospective cohort study | Lymphatic | Delayed LVA after VLNT for lower limb lymphedema |
| 2007 | Kim JS et al. | 24 | 1.26 | Case report | Reconstructive | Supermicrosurgical replantation of an amputated tongue |
| 2010 | Kim S et al. | 24 | 1.5 | Case report | Reconstructive | Supermicrosurgical replantation of an amputated nasal segment |
| 2019 | Phillips GSA et al. | 24 | 3.43 | Case series | Lymphatic | LVA for secondary lower limb lymphedema after cancer treatment |
| 2003 | Koshima I et al. | 23 | 1 | Case report | Reconstructive | Short-pedicle gluteal artery perforator flap using perforator-to-perforator anastomosis |
| 2017 | Yamamoto T et al. | 23 | 2.56 | Case report | Lymphatic | Deep lymphatic vessel-to-vein anastomosis for severe breast cancer-related arm lymphedema |
| 2025 | Hong JP et al. | 22 | 22 | Review / hypothesis article | Broad/conceptual | Proposed role of lymphatic supermicrosurgery in Alzheimer’s disease |
| 2022 | Scaglioni MF et al. | 22 | 5.5 | Prospective case series | Lymphatic | Combined superficial and deep LVA for refractory lymphedema |
| 2020 | Yamamoto T et al. | 20 | 3.33 | Technical review | Lymphatic | Technical pearls for lymphatic supermicrosurgery |
| 2023 | Escandón JM et al. | 20 | 6.67 | Systematic review/meta-analysis | Reconstructive | Systematic review of supermicrosurgical free flap transfer for soft tissue reconstruction |
| 2021 | Yang JC et al. | 19 | 3.8 | Prospective cohort study | Lymphatic | LVA-associated changes in oxidative stress and antioxidant capacity in lymphedema |
| 2023 | Onoda S et al. | 19 | 6.33 | Technical/educational article | Lymphatic | Standardised technical protocol for lymphaticovenular anastomosis |
| 2014 | Yamamoto T et al. | 18 | 1.5 | Case series | Technology-enabled | ICG-guided lymphaticolymphatic and lymphaticovenular anastomosis for iatrogenic lymphorrhea |
| 2018 | Pafitanis G et al. | 18 | 2.25 | Systematic review | Technology-enabled | Evidence-based simulation curriculum for supermicrosurgery training |
| 2022 | Mohos B et al. | 18 | 4.5 | Retrospective descriptive study | Technology-enabled | High-frequency ultrasound mapping for optimal LVA site selection |
| 2015 | Yamamoto T et al. | 18 | 1.64 | Case series | Lymphatic | Vein graft–assisted multisite LVA for uterine cancer-related lower limb lymphedema |
| 2009 | Koshima I et al. | 16 | 0.94 | Case report | Nerve | Cross-face supramicrosurgical nerve transfer for trigeminal branch II palsy |
| 2021 | Chen WF et al. | 16 | 3.2 | Narrative review / expert opinion | Lymphatic | Advanced technical pearls for successful LVA |
| 2022 | Hayashi A et al. | 16 | 4 | Review | Technology-enabled | Ultra-high frequency ultrasound for reconstructive and lymphatic supermicrosurgery |
| 2015 | Ayestaray B et al. | 16 | 1.45 | Case report | Technology-enabled | Fluorescein-guided lymphangiography for lymphatic supermicrosurgery |
| 2022 | Hong JP et al. | 15 | 3.75 | Review / technical article | Technology-enabled | Duplex ultrasound for recipient vessel selection in supermicrosurgery |
| 2022 | Caretto AA et al. | 15 | 3.75 | Retrospective cohort study | Lymphatic | Triple-incision LVA approach for early gynecological cancer-related lower limb lymphedema |
| 2009 | Yap YL et al. | 14 | 0.82 | Case report | Technology-enabled | Patent blue dye-assisted lymphatic mapping for LVA |
| 2021 | Will PA et al. | 14 | 2.8 | Case report with survey | Technology-enabled | 3D digital hybrid visualization-assisted LVA to improve ergonomics |
| 2020 | Yamamoto T et al. | 14 | 2.33 | Review | Broad/conceptual | Overview of supermicrosurgery for oncologic reconstruction |
| 2022 | Bianchi A et al. | 14 | 3.5 | Retrospective cohort study | Lymphatic | Recipient venule selection and anastomosis algorithm based on 1,000 LVAs |
| 2019 | Brebant V et al. | 13 | 1.86 | Case series | Technology-enabled | Augmented reality ICG-guided simplified LVA |
| 2022 | Yang JC et al. | 13 | 3.25 | Retrospective cohort study | Lymphatic | Propensity-matched analysis of retrograde anastomosis during LVA |
| 2012 | Mihara M et al. | 13 | 0.93 | Case report | Lymphatic | LVA for radical treatment of large pelvic lymphocyst |
| 2017 | Song D et al. | 13 | 1.44 | Retrospective case series | Reconstructive | Innervated dorsoradial perforator free flap for fingertip reconstruction |
| 2000 | Koshima I et al. | 13 | 0.5 | Case report | Reconstructive | Free paraumbilical perforator adiposal flap for breast reconstruction |
| 2024 | Burns HR et al. | 13 | 6.5 | Review | Technology-enabled | Review of robotics in microsurgery and supermicrosurgery |
| 2013 | Lasso JM et al. | 12 | 0.92 | Technical report / expert opinion | Lymphatic | Practical technical solutions for LVA |
| 2021 | Messa CA et al. | 12 | 2.4 | Observational anatomical study | Reconstructive | SCIP flap anatomical characterisation for free flap reconstruction planning |
| 2022 | Will PA et al. | 12 | 3 | Systematic review protocol | Lymphatic | Network meta-analysis protocol comparing LVA, VLNT, and conservative therapy |
| 2020 | Pinto V et al. | 11 | 1.83 | Experimental simulation study | Technology-enabled | 3D exoscope-assisted microvascular anastomosis on latex vessel models |
| 2014 | Iida T et al. | 11 | 0.92 | Case report | Reconstructive | Sensate ICAP flap for plantar reconstruction |
| 2015 | Lim SY et al. | 11 | 1 | Retrospective case series | Reconstructive | End-to-patch anastomosis for free flaps with small pedicles |
| 2021 | Scaglioni MF et al. | 11 | 2.2 | Retrospective case series | Reconstructive | Perforator-to-perforator free tissue transfer for defects around the body |
| 2023 | Yamamoto T et al. | 11 | 3.67 | Case series | Lymphatic | Office-based outpatient LVA under local anaesthesia |
| 2013 | Nuri T et al. | 10 | 0.77 | Technical report / case report | Lymphatic | Preparatory intravascular stenting technique for easier LVA |
| 2014 | Yoshimatsu H et al. | 10 | 0.83 | Technical report / case report | Lymphatic | Guide wire method for easier side-to-end LVA |
| 2019 | Yoshida S et al. | 10 | 1.43 | Comparative study | Lymphatic | Mechanical dilation using nylon monofilament to improve multisite LVA quality |
| 2024 | Reilly FOF et al. | 10 | 5 | Prospective implementation case series | Technology-enabled | Implementation of robot-assisted LVA using MUSA-2 |
| 2019 | Koshima I et al. | 10 | 1.43 | Case series | Reconstructive | Fingertip replantation techniques under digital block |
| 2015 | Yamamoto T et al. | 10 | 0.91 | Technical report / correspondence | Technology-enabled | Hands-free vein visualizer for recipient vein selection in LVA |
| 2016 | Yamamoto T et al. | 10 | 1 | Case report | Lymphatic | LVA for breast lymphedema after vascularized axillary lymph node transfer |
| 2023 | Yamamoto T | 10 | 3.33 | Case series | Reconstructive | Day-case free flap transfer as ambulatory supermicrosurgery |
| 2015 | Seo SW et al. | 10 | 0.91 | Case series | Reconstructive | Peroneal perforator flap for lower limb reconstruction |
| 2022 | Orfahli LM et al. | 9 | 2.25 | Narrative review / expert opinion | Lymphatic | Vascularized lymph vessel transplant and lymphedema treatment algorithm |
| 2024 | Lin CH et al. | 9 | 4.5 | Narrative review | Lymphatic | Overview of LVA efficacy, safety, imaging, technical refinement, and perioperative management |
| 2017 | Vijayan R et al. | 9 | 1 | Case report | Reconstructive | Venous supercharging of reverse-flow angular artery perforator flap for nasal reconstruction |
| 2013 | Gennaro P et al. | 9 | 0.69 | Case report | Nerve | Mandibular nerve fascicular cross-face transfer for sensory recovery after mandibulectomy |
| 2024 | Brahma B et al. | 9 | 4.5 | Retrospective cohort study | Lymphatic | Immediate lymphatic reconstruction using LVA ± LLA for breast cancer-related lymphedema prevention |
| 2023 | Tanna N et al. | 9 | 3 | Narrative review | Technology-enabled | Robotic breast surgery continuum including robotic-assisted supermicrosurgery |
| 2023 | Pereira N et al. | 8 | 2.67 | Retrospective observational study | Lymphatic | Algorithmic post-traumatic lymphedema treatment using LVA and SCIP lymphatic vessel flap |
| 2022 | Yang JC et al. | 8 | 2 | Retrospective cohort study | Lymphatic | LVA for secondary lower limb lymphedema in older patients |
| 2023 | Zhu J et al. | 8 | 2.67 | Case report | Technology-enabled | CEUS microbubble lymphography for LVA planning in severe lower limb lymphorrhea |
| 2019 | Wang KL et al. | 8 | 1.14 | Case report | Reconstructive | Split tibial flap from second toe for |
| 2018 | Ayestaray B | 7 | 0.88 | Case report | Lymphatic | LVA for male breast lymphoedema with clinical and MRI-confirmed volume reduction |
| 2019 | Yoshida S | 7 | 1 | Technical report / case series | Broad/conceptual | Mechanical dilation with nylon monofilament enabling ultra-small vessel anastomosis |
| 2023 | Banerjee A | 7 | 2.33 | Narrative review | Broad/conceptual | Overview of the history, current applications and future directions of supermicrosurgery |
| 2024 | Bailey EA | 7 | 3.5 | Narrative review / expert opinion | Lymphatic | Review of lymph node-to-vein anastomosis as an advanced lymphatic supermicrosurgical technique |
| 2024 | Mori F | 7 | 3.5 | Retrospective case series | Technology-enabled | Robotic-assisted micro/supermicrosurgical anastomosis in orthoplastic reconstruction |
| 2024 | Zheng Y | 7 | 3.5 | Pilot prospective case series | Lymphatic | Overlapping lockup LVA technique to address vessel size mismatch |
| 2017 | Yamamoto T | 7 | 0.78 | Case report | Reconstructive | Free prepuce perforator flap for dorsal finger defect |
| 2024 | Lo Torto F | 7 | 3.5 | Systematic review and meta-analysis | Reconstructive | Perforator-to-perforator anastomoses for lower-limb reconstruction |
| 2024 | Hayashi K | 7 | 3.5 | Technical note / expert opinion | Reconstructive | Technical guidance for fingertip replantation using supermicrosurgery |
| 2023 | Zheng L | 6 | 2 | Retrospective case series | Reconstructive | Free flaps with supermicrosurgery for maxillofacial oncological reconstruction |
| 2024 | Awad L | 6 | 3 | Systematic review | Technology-enabled | Robotic-assisted flap reconstruction and microsurgery across clinical, preclinical and educational settings |
| 2023 | Zhou X | 6 | 2 | Prospective comparative study | Technology-enabled | ICG lymphography plus methylene blue guidance for lower-limb LVA |
| 2020 | Arié A | 5 | 0.83 | Review article | Lymphatic | Diagnosis, evaluation and treatment of melanoma-related lymphoedema including LVA |
| 2002 | Allen RJ | 5 | 0.21 | Narrative review / historical overview | Reconstructive | Historical evolution of perforator flaps toward supermicrosurgery |
| 2024 | Knoz M | 5 | 2.5 | Retrospective propensity-score matched cohort study | Lymphatic | Comparison of contraction-type versus noncontraction-type lymphatic vessels in LVA |
| 2023 | Lin J | 5 | 1.67 | Retrospective case series | Reconstructive | Supermicrosurgical replantation of amputated facial tissues |
| 2025 | Sørensen RBA | 5 | 5 | Case series / preliminary observational study | Technology-enabled | Symani robotic system for microsurgical free flap procedures |
| 2019 | Iida T | 5 | 0.71 | Retrospective case series | Reconstructive | Vasa recta supermicrosurgical anastomosis for double-pedicled free jejunum transfer |
| 2023 | Hara H | 5 | 1.67 | Retrospective cohort study | Technology-enabled | Ultrasound lymphatic refill assessment and lymphatic washout after LVA |
| 2017 | Tashiro K | 5 | 0.56 | Case series | Broad/conceptual | Hemi-intravascular stenting technique for supermicrosurgical anastomosis |
| 2018 | Jeong HH | 4 | 0.5 | Case report | Oncological reconstruction | Supermicrosurgical helical composite free flap for nasal alar defect reconstruction after oncologic excision |
| 2023 | Pamuk Ç | 4 | 1.33 | Retrospective cohort study | Trauma | Supermicrosurgical replantation of Tamai Zone I fingertip amputations with functional and sensory outcome assessment |
| 2020 | Kristiansen M | 4 | 0.67 | Prospective cohort study | Lymphatic disease | Patient selection and outcome predictors for lower-limb lymphaticovenular anastomosis |
| 2024 | Frosolini A | 4 | 2 | Retrospective comparative study | Technology-enabled | 3D exoscope-assisted lymphatic supermicrosurgery compared with conventional microscope |
| 2014 | Yoon CS | 4 | 0.33 | Case series | Reconstructive | Posterior interosseous artery perforator free flap for hand and foot soft-tissue defects |
| 2023 | Seki Y | 4 | 1.33 | Retrospective case series | Lymphatic disease | Dynamic LVA for breast cancer-related lymphoedema without compression therapy |
| 2025 | Brown H | 4 | 4 | Systematic review | Technology-enabled | Review of robotic-assisted supermicrosurgery across lymphatic, reconstructive and nerve applications |
| 2018 | Visconti G | 4 | 0.5 | Narrative review | Lymphatic disease | Review of LVA and lymph node flap transfer for breast cancer-related peripheral lymphoedema |
| 2025 | Zurbuchen EA | 4 | 4 | Narrative review | Lymphatic disease | Modern review of lymphatic surgery including lymphovenous bypass and vascularized lymph node transfer |
| 2018 | El Fahar MH | 3 | 0.38 | Case report | Reconstructive | Paediatric intraoral reconstruction using free ALT flap and supermicrosurgery after corrosive injury |
| 2022 | Zhang G | 3 | 0.75 | Case report | Reconstructive | SCIP flap with intraoral anastomosis for palatal fistula reconstruction |
| 2020 | Carrasco-Lopez C | 3 | 0.5 | Anatomical observational study | Reconstructive | SCIP flap venous anatomy study for high-risk extremity reconstruction |
| 2019 | Gennaro P | 3 | 0.43 | Retrospective case series | Lymphatic disease | Ultramicrosurgical lymphatico-venular anastomosis for primary male genital lymphoedema |
| 2025 | Kiew CYK | 3 | 3 | Systematic review | Technology-enabled | AI-assisted training tools for microsurgery and supermicrosurgery education |
| 2019 | Koshima I | 3 | 0.43 | Case series / subgroup analysis | Lymphatic disease | Effect of pregnancy after multisite LVA for lower-limb lymphoedema |
| 2025 | Kaciulyte J | 3 | 3 | Case series | Lymphatic disease | LVA for facial lymphoedema after head and neck tumour resection |
| 2024 | Metz AA | 3 | 1.5 | Retrospective case series | Technology-enabled | ICG lymphography-guided microsurgery for lymphatic fistula treatment |
| 2024 | Wang W | 2 | 1 | Case series | Reconstructive | SCIP flap with intraoral anastomosis or pedicle elongation for maxillary defects |
| 2024 | MacKenzie A | 2 | 1 | Systematic review and meta-analysis | Reconstructive | Supermicrosurgery in craniofacial and head-and-neck soft tissue reconstruction |
| 2025 | Chen WF | 2 | 2 | Case series | Technology-enabled | Robot-assisted lymph node-to-vein anastomosis in 22 cancer-related lymphoedema cases |
| 2025 | Chang C | 2 | 2 | Prospective cohort study with propensity score matching | Lymphatic disease | Groin-only LVA compared with conventional distal/multi-incision LVA for lower-extremity lymphoedema |
| 2023 | Gennaro P | 2 | 0.67 | Retrospective case series | Lymphatic disease | Supramicrosurgical lymphatico-venular anastomosis for recurrent lymphatic fistulas |
| 2026 | Lahmar T | 2 | 2 | Systematic review | Other / CNS lymphatic dysfunction | Systematic review of brain lymphatic drainage and deep cervical lymphatic surgery for neurodegenerative disease |
| 2025 | Hinson C | 2 | 2 | Systematic review and meta-analysis | Lymphatic disease | Immediate lymphovenous anastomosis to prevent secondary lymphoedema after oncologic lymphadenectomy |
| 2020 | Biermann N | 2 | 0.33 | Comparative study | Technology-enabled | PEMF therapy as an adjunct during supermicrosurgical lymphovenous anastomosis to influence lymphatic flow |
| 2025 | Jeong YJ | 2 | 2 | Systematic review | Technology-enabled | Systematic review of Symani robotic system feasibility and applications in micro/supermicrosurgery |
| 2025 | Hashem T | 2 | 2 | Retrospective cohort study | Lymphatic disease | Ultrasound-guided LVA for breast cancer-related lymphoedema in low-resource settings |
| 2018 | Cereceda-Monteoliva N | 1 | 0.13 | Case report with narrative review | Reconstructive | Supermicrosurgical repair of small-calibre vascular pedicle to salvage DIEP free flap after pedicle avulsion |
| 2024 | Peng P | 1 | 0.5 | Retrospective case series | Trauma | Supermicrosurgical digit-tip replantation after crush injury with functional and sensory outcomes |
| 2024 | Kim HB | 1 | 0.5 | Retrospective comparative study | Reconstructive | Comparison of small-vessel <0.8 mm and larger-vessel free flap anastomoses for perfusion adequacy |
| 2011 | Iannetti G | 0 | 0 | No abstract | Reconstructive | Surgical treatment of inferior alveolar nerve deficit |
| 2024 | Poskevicius A | 1 | 0.5 | Retrospective case series | Oncological reconstruction | Combined perforator flap reconstruction and lymphatic procedures after sarcoma resection |
| 2023 | Liang WH | 1 | 0.33 | Retrospective cohort study | Technology-enabled | Preoperative ultrasound mapping for LVA planning in advanced breast cancer-related lymphoedema |
| 2021 | Khajuria A | 1 | 0.2 | Retrospective comparative case series | Reconstructive | Use of arborization capture and venous supercharging to improve free-styled propeller flap survival |
| 2024 | Zhou Z | 1 | 0.5 | Case report | Lymphatic disease | Rescue LVA for ulcers and lymphorrhea after Charles procedure |
| 2025 | Bustos SS | 1 | 1 | Retrospective diagnostic comparison study | Technology-enabled | CEUS versus ICG lymphography for lymphatic mapping before LVA |

Supplementary Table 1. Citation Profile and Principal Contributions of Included Supermicrosurgery Studies
